# Toothbrush: A Report of an Unusual Foreign Body

**Published:** 2015-05

**Authors:** Mohammad Reza Farahnak, Somayeh Araghi, Soheila Nikakhlagh, Nader Saki

**Affiliations:** 1 *Hearing and Speech Research Center, Ahvaz Jundishapur University of Medical Sciences, Ahvaz, Iran.*

**Keywords:** Pharynx, Unusual Foreign body, Toothbrush

## Abstract

**Introduction:**

Ingestion of a foreign body is a common problem among all age groups. Most of the foreign bodies in the pharynx are usually lodged at the level of cricopharynx. The diagnosis is based on history, clinical, and radiological examination. Most foreign-body ingestions are accidental, but there may be contributory factors such as mental disorder, alcoholism, and prison incarceration. Toothbrush ingestion is uncommon and requires prompt medical attention.

**Case Report:**

In this article, a rare case of a toothbrush foreign body is presented. The ingestion was caused by a seizure and the toothbrush was removed through surgical management.

**Conclusion:**

An ingested toothbrush will not pass spontaneously. The best management is early endoscopy performed by a skilled surgeon. If this is unsuccessful, surgical management can be performed.

## Introduction

Ingestion of a foreign body is a common problem among all age groups, particularly in infants and children as they have a tendency to put anything in their mouth, which may cause accidental ingestion in the aerodigestive system. Objects may get lodged in the tonsil, base of tongue, piriform fossae, and esophagus or sometimes in the larynx or lower down in the respiratory tract leading to medical/surgical emergencies, which are often challenging to an otolaryngologist. Most of the foreign bodies in the pharynx are usually lodged at the level of cricopharynx or lower down in the esophagus or in the airway in the right bronchus when the larynx is small. Occasionally foreign bodies lodged in the larynx may be fatal. The diagnosis is based on history, clinical, and radiological examination (1,8). Depending on the age, a vast variety of foreign bodies like coins, marbles, buttons, batteries, bottle tops, peas, beans, grain, sand, and seeds are more often seen in infants and children, while bones, dentures, and metallic pins/wires have been reported more often in adults. In addition, fish bone is perhaps the most common foreign body in the pharynx reported in the adult population (1).

Most foreign-body ingestions are accidental, but there may be contributory factors such as mental disorder, alcoholism, and prison incarceration. Toothbrush ingestion is uncommon and requires prompt medical attention. Although 80% of ingested foreign bodies pass spontaneously, some of them require endoscopic or surgical removal (2). In this article, a rare case of a toothbrush foreign body is presented. The ingestion was caused by a seizure and the toothbrush was removed through surgical management.

## Case Report

**Fig 1:**
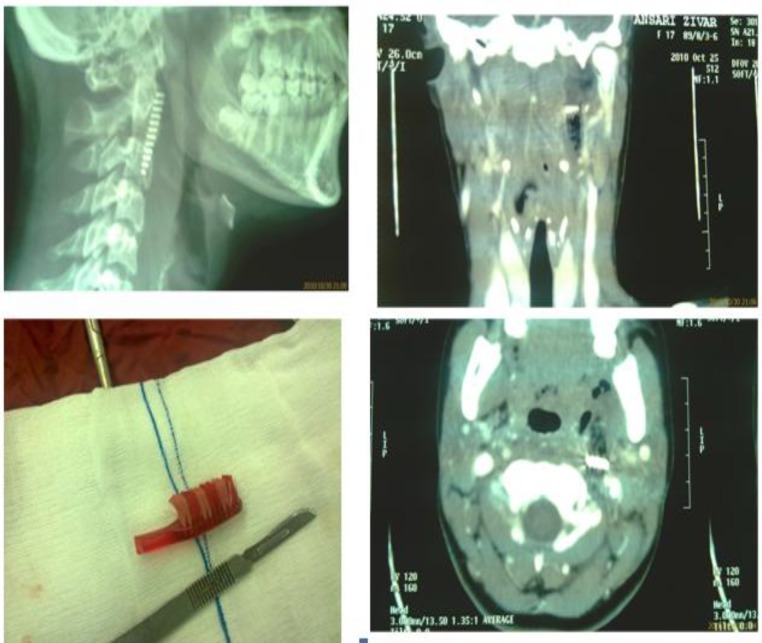
Tooth brush ingested as a foreign body

This is a case report of a 17-year-old girl who accidentally swallowed a part of her toothbrush. This patient presented herself in the emergency room with pain, anterior neck tenderness, and odinophagia. History revealed that she had a seizure while brushing her teeth and the head of the toothbrush broke in the oral cavity. She had normal vital signs. Examination showed a 1.5 cm left retromolar laceration exactly anterior to the anterior tonsillar pillar in the oral cavity without any hematoma or active bleeding. A lateral neck X-ray revealed the bristled part of the toothbrush and a neck CT scan showed it as well. Under general anesthesia, exploration of the parapharynx space via the intraoral laceration was performed but the foreign body wasn’t detected. Rigid esophagoscopy was performed as well but the same result was achieved. Therefore left lateral neck exploration was performed and the foreign body was removed (Fig.1). The intraoral laceration was repaired. Subsequent recovery was uneventful.

## Discussion

Toothbrush ingestion is uncommon and requires prompt medical attention(3). In a case report in 2012 by Sew Paul and colleagues, a swallowed toothbrush passed past the pylorus and perforated the terminal ileum (4). In another case report in 2013 by Karim Jamal and colleagues, the toothbrush became partially embedded in the gastric mucosa (5). In this case, the toothbrush head was in the parapharyngeal space. 

In an article in 1994, Duncan and colleagues presented 2 cases of toothbrush ingestion by bulimics (6). In 2006, Jan Christoph Karcher and colleagues reported toothbrush ingestion in a patient with mental retardation (infantile cerebral palsy and psychomotor retardation) (7). In this case, the patient had a seizure while brushing her teeth. In several cases and in this case, surgical management was performed in order to remove the foreign body (3-7); however, in some cases, the toothbrush was extracted under local anesthesia using a fiber-optic endoscope (8).

## Conclusion

An ingested toothbrush will not pass spontaneously and has a significant risk of causing pressure necrosis or perforation, which can result in life-threatening sepsis. Unlike smaller foreign bodies within the stomach, a trial of conservative therapy should not be employed. The best management is early endoscopy by a skilled surgeon. If this is unsuccessful surgical management can be performed.
